# Supplementary material for: A distributed framework for zero-day malware detection using federated ensemble models
Source: PLoS One. 2026 Jan 7;21(1):e0339907. doi: 10.1371/journal.pone.0339907 (PMC12779155; doi:10.1371/journal.pone.0339907)
Supplement: Attachment — Submitted filename: major revision PONE-D-25-34169.docx [file pone.0339907.s002.docx]

**major revision comments** for the PLOS ONE manuscript *“A Distributed Framework for Zero-Day Malware Detection Using Federated Ensemble Models”*.

**1. Novelty and positioning are unclear**

The proposed Ensemble Federated Model is described as “novel,” but federated ensemble methods for malware detection already exist. Authors must:

- Explicitly state what is **technically new** (stacking method, weighting scheme, architecture).
- Compare with the latest FL-based malware detection frameworks and clarify how this differs.
- Strengthen the contribution list to highlight unique innovations.

**2. Methodology lacks reproducibility**

Equations (1)–(20) provide generic math (binary conversion, CNN layers, weighted averaging) but not the actual **fusion or stacking algorithm**. Revise methodology to:

- Provide detailed pseudocode for training, communication, and stacking steps.
- Clarify how local models’ predictions are combined (majority voting, weighted ensemble, meta-learner?).
- Define hyperparameters (epochs, batch size, optimizer, learning rate).

**3. Dataset description is insufficient**

The manuscript uses both a **private dataset (19,600 PE files)** and Malimg dataset but:

- Does not specify how PE binaries were collected, cleaned, and validated.
- Provides no labeling procedure details (who annotated, which antivirus outputs were trusted).
- Fails to mention dataset balance, augmentation strategy, or data leakage prevention.
  → A **dataset preparation flowchart** is needed.

**4. Baseline selection and fairness are weak**

Comparisons are limited to AlexNet, ResNet-18, and ResNet-50. To make claims valid:

- Add strong modern baselines: EfficientNet, DenseNet, Vision Transformers, federated averaging (FedAvg).
- Ensure identical preprocessing and training conditions for fair benchmarking.
- Provide ablations: single ResNet vs. ensemble vs. federated ensemble.

**5. Metrics and evaluation issues**

Metrics like “accuracy, precision, recall, F1” are used, but standard **malware detection benchmarks** require:

- ROC curves and AUC across thresholds.
- Confusion matrices per class (not just node-level).
- Robustness measures (false positive rate, detection delay, runtime cost).
  Also, “FRN” is undefined and inconsistent.

**6. Zero-day detection claim needs evidence**

The manuscript claims improved “zero-day” detection, but experiments simply test on held-out families.

- Authors should design **leave-one-family-out** evaluation to mimic unseen families.
- Provide detection rates across multiple random seeds for robustness.
- Clarify how the model adapts when **truly novel families** are encountered.

**7. Federated learning privacy and communication cost not discussed**

The model is framed as “privacy-preserving,” but:

- There is no analysis of communication overhead (number of rounds, bandwidth).
- No mention of potential leakage from gradients or model parameters.
- Add discussion of differential privacy or secure aggregation to justify privacy claims.

**8. Figures and tables need overhaul**

- Figures 1–6 are generic diagrams; more **technical illustrations** are needed (federated workflow, stacking layer, data pipeline).
- Tables 2–8 are overloaded with raw results but lack proper captions and context.
- Add plots of **training/validation curves**, **class-wise bar charts**, and **latency vs accuracy** graphs.

**9. Literature review is outdated and poorly curated**

The Related Work section cites older CNN studies while missing **recent FL and ensemble malware detection papers (2022–2024)**. Authors should:

- Incorporate IoT-specific FL malware studies, lightweight models, and graph neural network approaches.
- Critically compare these with the proposed method.
- Remove tangential works (e.g., tuberculosis CNN feature extractors).

**10. Writing quality and consistency issues**

- Grammar and flow need editing: e.g., phrases like *“this situation significant security risks”* are ungrammatical.
- The paper inconsistently refers to **ResNet-18** vs. **ResNet-50** as feature extractors.
- Abstract says “ResNet-18,” contributions list “ResNet-50.” Must resolve contradictions.
- Overuse of filler phrases (e.g., “robust,” “intelligent,” “novel”) without evidence.
