# Supplementary material for: A distributed framework for zero-day malware detection using federated ensemble models
Source: PLoS One. 2026 Jan 7;21(1):e0339907. doi: 10.1371/journal.pone.0339907 (PMC12779155; doi:10.1371/journal.pone.0339907)
Supplement: Attachment — Submitted filename: Responce to Reviewer.docx [file pone.0339907.s003.docx]

**A Distributed Framework for Zero-Day Malware Detection Using Federated Ensemble Models**

**Point by Point Response Reviewer 1**

**Comment 1:** In paper their claimed contribution is not sufficiently distinguished from existing malware-detection approaches. The authors should clarify what is genuinely new beyond applying standard federated learning with an ensemble.

**Our Response:**

Thank you for your comment. We appreciate the reviewer’s suggestion. We have restructured the key contributions section to highlight clear technical points

II. CONTRIBUTION

The main contribution of this proposed research is to present a robust malware families classification approach that improve accuracy in zero-day attacks and inter and intra-classification using ensemble federated learning framework. The key contributions of the proposed work include the following:

1)	A novel stacked ensemble federated model is proposed for robust malware classification. In this framework, features extracted from different distributed nodes are combined and their outputs are fused through a central ensemble. By fusing both node-level and global level prediction, model improves classification performance and generalization as compare with traditional multi-node fusion techniques.

2)	Fine-tuned ResNet-50 model with transfer learning is employed to extract features from PE files. These features are then converted into image representation that are utilized within federated pipeline to reduce training cost while preserving classification accuracy.

3)	This model is designed to address two major challenges: class imbalance and high structural similarity and complexity of real-time malware classification through accuracy-aware dynamic weighting technique that efficiently adjust predictions across different malware families than conventional methods for class imbalance.

4)	The proposed model improved performance in malware classification not only from federated learning but also from integration of ensemble stacking with distributed architecture.

**Comment :** The description of the ensemble federated learning mechanism in proposed methodology is brief and lacks sufficient technical explanation and motivation.

**Our Response:**

Thank you for your keen concentration. The changes have been including under the proposed methodology section as:

C. ENSEMBLE FEDERATED MODEL

A distributed network is designed in which multiple entities, nodes, or devices work together to detect malware while retaining a certain degree of control over their local repository. This allows model to be trained across distributed devices without learning raw data, ensuring privacy. The central server initially computes the average of the received model parameters, producing a global average model as a combination of the local models. This training is repeated over multiple epochs to refine the global model. However, simple parameter averaging (as in FedAvg) is not sufficient because data across node is highly imbalanced as it dominates major malware families while minority families remain under-represent. Therefore, in this case pure averaging with hierarchical stacking ensemble mechanism that integrated level predictions through meta-learners and weight each node according to its accuracy, this amplifies noise from weaker nodes and ensure reliable node contribution for model decision.

**Comment :** The paper does not specify the exact train/validation/test split or the number of cross-validation folds, making it difficult to assess robustness. There must be clearly stated Key training parameters such as learning rate, batch size, optimizer, number of epochs)

**Our Response:**

Thank you for your comment. We have expanded the section with data splitting method and specific hyperparameters as:

The dataset is split into three subsets 70 % for training, 15% for validation and 15% for testing. To ensure the robustness of propose model key training hyperparameters are selected such as 0.001 learning rate with Adam optimizer over 50 epochs and 32 batch size and experiments were also repeated using 5-fold cross validation with Cross-entropy as an objective loss function.

Communication between nodes and server over a local network with synchronous updates in each round. Each round utilized five local epochs per node before the server performed aggregation. Overall, 20 communication rounds were required where each node exchange about 24 MB parameters per update and total cost across 20 rounds was approximate 1.9 GB for just 4 nodes. In this communication raw data is not shared because its loss some gradients and parameters information. To overcome this model, integrate differential privacy (DP) or secure aggregation (SA) protocols are applied that ensures results reproducibility and scalability to real malware detection tasks.

**Comment :** Although zero-day detection is mentioned in the article, experimental evidence for this scenario is not described in detail to evaluate its validity.

**Our Response:** Thank You for this valuable suggestion the now to evaluate the effectiveness of Zero-day detection changes are made under the section as:

EXPERIMENT 4: ZERO-DAY ATTACK PERFORMANCE

This experiment is conducted to evaluate the strength of the model against zero-day attacks, which are new malware threats that cannot be detected easily. To stimulate real conditions, some malware families such as Graftor, Kazy, and BrowseFox (Adware), Zbot and Agent (Trojan), and Mydoom (Worm) were not included in training but used as unseen malware during testing. This ensures the model’s efficiency results can detect unseen threats rather than simple recognition of known families.

Table 6 shows the behavior of different malware families and the performance of various deep learning pretrained models against these attacks. The table includes similar performance measures along with the detection rate, which shows the percentage of correctly identified malware samples for each model.

Experimental results show that among all traditional deep learning models, proposed ensemble models perform better and achieve the highest overall performance with 94.6% detection rate, 0.91 F1-Score that highlighting the importance of integrating multiple models to defend against zero-day attacks. This improvement is achieved from the hierarchical stacking method, which utilized node diversity and integrate predictions from multiple nodes. This experiment also supports the model to handle new malware variants and demonstration stronger zero-day detection than standard federated averaging.

**Comment**: The comparative analysis uses only a limited set of baselines; more recent deep or hybrid architectures should be included with confidence intervals or significance tests are provided to substantiate the reported performance improvements.

**Our Response:**

Thank you for your keen concentration. We have revised the manuscript accordingly

- In addition to AlexNet, ResNet-18, and ResNet-50, we now include EfficientNet-B0, DenseNet-121, Vision Transformer (ViT-B/16), and a FedAvg baseline. These were trained under identical preprocessing and evaluation conditions to ensure fairness. The results are reported in Table 3 (Cross-Dataset Generalization).
- The proposed stacked federated ensemble is compared against these recent architectures, clearly showing that while EfficientNet, DenseNet, and ViT achieve competitive accuracy, our ensemble achieves superior overall performance (95.4 ± 0.2% accuracy, F1 = 0.93, AUC = 0.97).
- Now all performance metrics in Table 3 are reported as mean ± standard deviation, computed over five independent runs with different random seeds. This provides confidence intervals and demonstrates that the reported improvements are consistent rather than due to chance.
- The table also includes model complexity measures (Params, FLOPs, inference latency) to contextualize accuracy gains. This highlights that ResNet-50 offers the best trade-off between performance and efficiency, and justifies its selection as the backbone for our proposed framework.

**Comment**: Detail about computational environment such as hardware requirement and federated setup (e.g., number of clients, network conditions) are must be included

**Our Response:**

Thank you for your comment. We appreciate your suggestion. These hardware requirements are added under the section Dataset Description after describing the dataset and additions are highlighted in manuscript also:

This experiment is conducted on an intel Core i9 processor with 64 GB RAM and NVIDIA RTX 3090 GPU (24 GB) in laboratory setting. The model implementation and federated setup is stimulated in Python 3.10 with PyTorch 2.0 with multiple client nodes each containing a subset of malware families on single workstation with central server. Communication between nodes and server over a local network with synchronous updates in each round. Each round utilized five local epochs per node before the server performed aggregation. Overall, 20 communication rounds were required where each node exchange about 24 MB parameters per update and total cost across 20 rounds was approximate 1.9 GB for just 4 nodes. In this communication raw data is not shared because its loss some gradients and parameters information. To overcome this model, integrate differential privacy (DP) or secure aggregation (SA) protocols are applied that ensures results reproducibility and scalability to real malware detection tasks.

**Comment**: Some variables in the equations (for example H, W, and C) are introduced without definition which leads towards ambiguity.

**Our Response:**

We thank the reviewer for pointing out this oversight. In the revised manuscript, we have carefully reviewed all equations and ensured that each variable is clearly defined at the point of introduction. Specifically:

Variables such as H (height), W (width), and C (number of channels) are now explicitly defined either immediately before or after the corresponding equations. These additions and clarifications have been highlighted in the revised version for easy identification.

**Comment** : The manuscript contains some grammatical issues; professional language editing is recommended.

**Our Response:**

We thank the reviewer for this observation. The revised manuscript has been thoroughly re-edited to correct grammatical errors, improve sentence flow, and ensure professional academic writing style. Careful attention was given to clarity, conciseness, and consistency throughout the paper.

**Comment** The Experiments and Results section would benefit from a concluding subsection that highlights the main findings.

**Our Response:**

Thank you for the insightful suggestion. In response, we have added a dedicated concluding subsection at the end of the Experiments and Results section titled “Key Findings and Insights.” This subsection summarizes the major experimental outcomes in a concise and comparative manner. Specifically, it highlights:

- The performance advantage of the proposed stacked ensemble federated learning model over traditional single and FedAvg models, achieving the highest detection accuracy (95.4%) and F1-score (0.93).
- The cross-dataset generalization capability when trained on the private malware dataset and tested on Malimg.
- The computational tradeoffs of different backbones (ResNet-18, ResNet-50, EfficientNet, ViT) in terms of FLOPs and inference time, justifying our selection of ResNet-50 for its balance of speed and accuracy.
- The model’s robustness to zero-day attacks, with detection rates exceeding 94.6% using both held-out and leave-one-family-out protocols.
- Newly added ROC-AUC curves, confusion matrices, and robustness metrics (false positive rate, communication cost) to align with standard malware detection benchmarks.

**Comment**: Provided source code, implementation instructions and dataset accessibility are provided without these, independent verification of the results is difficult. A link or clear guidance for requesting the material should be included in the supplementary material.

**Our Response:**

Thank you for your comment. We revised and added a clear link <https://github.com/jhshah101/Zero-Day-Malware-Detection>, which is highlighted in the manuscript.

**Reviewer 2**

**Comment** 1: Novelty and positioning are unclear

The proposed Ensemble Federated Model is described as “novel,” but federated ensemble methods for malware detection already exist. Authors must:

•	Explicitly state what is technically new (stacking method, weighting scheme, architecture).

•	Compare with the latest FL-based malware detection frameworks and clarify how this differs.

•	Strengthen the contribution list to highlight unique innovations.

**Our Response:**

We thank the reviewer for pointing out the need to clarify the novelty and positioning of our work. In the revised manuscript, we have made the following changes:

- The abstract and contribution section have been revised to explicitly state our unique innovations, including (i) the stacking-based federated ensemble design, (ii) integration of diverse node-level predictions for robustness, and (iii) evaluation on a large-scale PE malware dataset with data distribution across multiple nodes.
- We have added a comparison with existing federated learning frameworks for malware detection in the related work section as mentioned in Table 1. To the best of our knowledge, prior studies applied traditional federated averaging (FedAvg) and simple ensemble fusion that cannot handle data distribution across different nodes and classes, as we do in this article.
- Figure 1 has been updated and made more prominent to illustrate the proposed stacking-based ensemble mechanism, highlighting how predictions from multiple federated nodes are combined through a logistic regression meta-learner rather than simple averaging.
- The conclusion has been revised to emphasize how our approach differs from standard FedAvg and previously published FL-based malware detection methods, and to highlight the practical value of our stacking-based design in handling real-world data distribution across nodes.

These additions clarify the novelty of our work, strengthen the contribution list, and clearly position our proposed model relative to prior federated ensemble approaches.

**Comment** 2: Methodology lacks reproducibility

Equations (1)–(20) provide generic math (binary conversion, CNN layers, weighted averaging) but not the actual fusion or stacking algorithm. Revise methodology to:

•	Provide detailed pseudocode for training, communication, and stacking steps.

•	Clarify how local models’ predictions are combined (majority voting, weighted ensemble, meta-learner?).

**Our Response:**

We are sincerely grateful to the reviewer for this insightful comment. In response, we have carefully revised and substantially expanded the methodology section to provide a clearer and more detailed description of the proposed workflow:

- Revised Section IV.C (Methodology): We added a clear description of the proposed federated ensemble workflow, explicitly detailing how training, communication, and stacking occur in our framework. Each client node independently trains a ResNet-50 model on its local dataset and transmits both the model weights and softmax prediction probabilities to the central server.
- Unlike traditional federated learning methods (e.g., FedAvg) that aggregate model parameters, or simple ensemble methods that rely on majority voting, our approach employs stacking. At the server, the softmax outputs from all clients are concatenated to construct a stacked feature matrix. A logistic regression classifier is then trained as a meta-learner on this matrix, enabling the global model to learn complementary decision boundaries across heterogeneous client nodes.
- To ensure transparency and reproducibility, we have added detailed pseudocode that explicitly outlines the training phase (local updates at clients), communication phase (model weights and predictions sent to the server), and stacking phase (meta-learner training at the server).
- Hyperparameters such as batch size, learning rate, optimizer choice, number of local epochs, and loss function are included in the pseudocode and also in data description section where we have also added the to make the workflow unambiguous and reproducible.

3. Dataset description is insufficient

The manuscript uses both a private dataset (19,600 PE files) and Malimg dataset but:

•	Does not specify how PE binaries were collected, cleaned, and validated.

•	Provides no labeling procedure details (who annotated, which antivirus outputs were trusted).

•	Fails to mention dataset balance, augmentation strategy, or data leakage prevention.

→ A dataset preparation flowchart is needed.

**Our Response:**

We thank the reviewer for this important comment. In the revised manuscript, we have substantially improved the dataset description and added the requested flowchart:

- We now specify that the private dataset consists of 19,600 Win32-type malware samples collected from multiple open repositories (GitHub, VirusShare, MalwareBazaar). Rigorous filtration was applied by computing MD5 and SHA256 hashes for all binaries, which were cross-verified through VirusTotal to obtain metadata, file type, and detection signatures, all changes are highlighted in manuscript.
- To ensure reliable ground-truth labels, we retained only samples flagged as malicious by at least three independent antivirus engines. Final family labels were standardized using F-Secure’s detection titles, resulting in 28 consistent classes for classification.
- All binaries and associated metadata were stored in MongoDB, preventing duplication across samples. To eliminate data leakage across subsets, a strict hash-based splitting strategy was applied. The dataset was divided into 70% training (14,000 samples), 15% validation (2,800 samples), and 15% testing (2,800 samples).
- Each malware binary was converted into a grayscale image and normalized to 255×255 pixels. Data augmentation was applied during validation to enhance generalization.
- To make the dataset creation pipeline transparent, we added Figure 4 (Dataset Creation Steps), which illustrates the collection, filtering, labeling, and preprocessing workflow.

4. Baseline selection and fairness are weak

Comparisons are limited to AlexNet, ResNet-18, and ResNet-50. To make claims valid:

•	Add strong modern baselines: EfficientNet, DenseNet, Vision Transformers, federated averaging (FedAvg).

•	Ensure identical preprocessing and training conditions for fair benchmarking.

Provide ablations: single ResNet vs. ensemble vs. federated ensemble.

**Our Response:**

We thank the reviewer for this constructive comment. In the revised manuscript, we have strengthened our baseline selection, ensured fair benchmarking, and added ablation studies as follows:

- In addition to AlexNet, ResNet-18, and ResNet-50, we now include EfficientNet-B0, DenseNet-121, Vision Transformer (ViT-B/16), and a FedAvg baseline (ResNet-18). The results are summarized in Table 3 (Cross-Dataset Generalization).
- To provide a fair assessment beyond accuracy, we report number of parameters, FLOPs, and inference time per image. This shows that while EfficientNet, DenseNet, and ViT achieve competitive accuracy, they require substantially higher FLOPs and inference time compared to ResNet-50, which balances accuracy (93.1%) with computational efficiency (4.1 GFLOPs, 7.9 ms/img).
- We have added an ablation experiment (Table 4) to demonstrate the incremental benefit of our proposed stacked federated ensemble over both a single ResNet-50 model and FedAvg (ResNet-50, parameter averaging). The results clearly show that:
- Single ResNet-50 achieves 93.1% accuracy (F1 = 0.91, AUC = 0.96),
- FedAvg slightly underperforms at 92.2% (F1 = 0.90, AUC = 0.95),
- Our proposed stacked federated ensemble achieves 95.4% accuracy (F1 = 0.93, AUC = 0.97).

Based on these results, we justify the choice of ResNet-50 as the backbone for our proposed framework.

5. Metrics and evaluation issues

Metrics like “accuracy, precision, recall, F1” are used, but standard malware detection benchmarks require:

•	ROC curves and AUC across thresholds.

•	Confusion matrices per class (not just node-level).

•	Robustness measures (false positive rate, detection delay, runtime cost).

Also, “FRN” is undefined and inconsistent.

**Our Response:**

Thank you for highlighting the need for comprehensive evaluation metrics. In response:

- We have included per-class confusion matrices in Figure 5, showing the detailed classification behavior for each malware group (Adware, Trojan, Worm, Win32), ensuring transparency and interpretability beyond node-level summaries.
- To meet the benchmark requirement of ROC analysis, we have added Figure 6, which presents ROC curves and AUC scores across thresholds for all malware families. This helps evaluate the model’s discrimination ability at varying thresholds, as per standard practice in malware detection.
- We now also report robustness measures:
- False Positive Rate (FPR) is derived from confusion matrices for each group.
- Detection latency and communication overhead are discussed in Section V under heading Dataset Description, along with hardware setup and runtime cost.

6. Zero-day detection claim needs evidence

The manuscript claims improved “zero-day” detection, but experiments simply test on held-out families.

•	Authors should design leave-one-family-out evaluation to mimic unseen families.

•	Provide detection rates across multiple random seeds for robustness.

•	Clarify how the model adapts when truly novel families are encountered.

**Our Response:**

We thank the reviewer for this valuable suggestion. In the revised manuscript, we have substantially strengthened the zero-day evaluation protocol as follows:

- In Section F, we now explain that our hierarchical stacking approach enables adaptation to unseen families by integrating diverse client-level predictions into a logistic regression meta-learner. This increases the likelihood of capturing shared structural patterns (e.g., PE headers, byte-image embeddings) across families, thereby improving resilience when encountering malware variants not present in training.
- First results are reported in Held-out family experiment retained Table 7 where selected families (e.g., Graftor, Kazy, BrowseFox from Adware; Zbot and Agent from Trojan; Mydoom from Worm) were excluded during training and treated as unseen at test time. This provides an intuitive scenario-driven test of the model’s ability to generalize beyond known families
- To validate zero-day detection, we implemented a leave-one-family-out (LOFO) protocol, where each malware family was excluded once during training and considered as unseen during testing. This procedure was repeated across all 28 families and averaged over five random seeds. The ensemble model achieved a 94.6 ± 0.4% detection rate and 0.91 ± 0.01 F1-score, demonstrating robustness and stability under unseen-family evaluation.
- By averaging results over five seeds, we confirmed that the model’s performance is not due to a single initialization or random split, but is consistent across multiple runs.

7. Federated learning privacy and communication cost not discussed

The model is framed as “privacy-preserving,” but:

•	There is no analysis of communication overhead (number of rounds, bandwidth).

•	No mention of potential leakage from gradients or model parameters.

•	Add discussion of differential privacy or secure aggregation to justify privacy claims.

**Our Response:**

We appreciate the reviewer’s thoughtful comment. In response, we have revised Section IV (Implementation Details) to include a detailed analysis of communication overhead and a clear discussion of privacy-related considerations.

- We now specify that our federated setup involves 20 communication rounds, where each client node sends approximately 24 MB of model parameters to the central server per round. With four nodes in total, the overall communication cost amounts to approximately 1.9 GB. Each round consists of five local training epochs per node, followed by synchronous aggregation at the server.
- While the proposed framework avoids raw data sharing between nodes, we acknowledge the potential for sensitive information leakage through shared gradients or model parameters. To address this, we have added a discussion outlining plans to integrate formal privacy-preserving mechanisms in future work.
- Specifically, we now mention the use of protocols such as Google’s RAPPOR, Apple’s Differential Privacy, and Secure Federated Aggregation (SFA) as used in Google’s Federated Learning deployments to mitigate risks such as gradient leakage and membership inference.

8. Figures and tables need overhaul

•	Figures 1–6 are generic diagrams; more technical illustrations are needed (federated workflow, stacking layer, data pipeline).

•	Tables 2–8 are overloaded with raw results but lack proper captions and context.

•	Add plots of training/validation curves, class-wise bar charts, and latency vs accuracy graphs.

**Our Response:**

We appreciate the reviewer’s feedback on the need to improve the clarity and informativeness of our figures and tables. In the revised manuscript, we made the following changes:

- Figure 1 has been redesigned to present a more detailed and technical illustration of the proposed federated stacking workflow for malware detection, clearly showing the client-server communication and stacking meta-learner.
- A new Figure 4 (Dataset Creation Steps) has been added to illustrate the complete data pipeline, including collection, filtration, labeling, and preprocessing.
- A new Figure 5 provides family-wise confusion matrices for Adware, Trojan, Worm, and Win32 groups, giving readers a granular view of class-level performance.
- Figure 6 now includes training and validation curves, enabling readers to evaluate convergence behavior and generalization.
- Tables 2–8 have been reformatted with clearer captions and added context, making them self-explanatory and easier to interpret.
- Performance metrics are reported in detail, including mean ± standard deviation across multiple seeds, and complexity measures (parameters, FLOPs, inference latency) are included for fairness.
- Class-level performance is presented in bar charts (confusion matrix visualization).
- Latency vs. accuracy comparisons is integrated into the results to highlight the trade-offs between performance and computational efficiency.

**Old Figure 1**:

**New Figure 1**:

9. Literature review is outdated and poorly curated

The Related Work section cites older CNN studies while missing recent FL and ensemble malware detection papers (2022–2024). Authors should:

•	Incorporate IoT-specific FL malware studies, lightweight models, and graph neural network approaches.

•	Critically compare these with the proposed method.

•	Remove tangential works (e.g., tuberculosis CNN feature extractors).

**Our Response:**

We thank the reviewer for highlighting the need to strengthen and update the literature review. In the revised manuscript, we have made the following changes:

- We incorporated several recent works on federated learning for malware detection, including IoT-specific frameworks, ensemble-based approaches, and lightweight models. These updates are reflected both in the Related Work section and in the new comparison summary (Table 2).
- We now also discuss recent graph neural network (GNN)-based approaches and lightweight CNN/Transformer models relevant to malware detection and IoT environments. This provides a more comprehensive context for our proposed method.
- The Related Work section has been revised to not only cite these studies but also critically compare them with our stacking-based federated ensemble. In particular, we highlight how most prior works either (i) apply federated averaging on homogeneous backbones, or (ii) focus on IoT datasets with limited class diversity, whereas our method addresses heterogeneity across multiple nodes and integrates predictions through a meta-learner for improved robustness.
- We acknowledge the reviewer’s observation that a tuberculosis CNN paper was mistakenly included. This was originally cited for its use of the VGG model, but we agree it was tangential to our study. It has now been replaced with a directly relevant malware detection reference.

10. Writing quality and consistency issues

•	Grammar and flow need editing: e.g., phrases like “this situation significant security risks” are ungrammatical.

•	The paper inconsistently refers to ResNet-18 vs. ResNet-50 as feature extractors.

•	Abstract says “ResNet-18,” contributions list “ResNet-50.” Must resolve contradictions.

•	Overuse of filler phrases (e.g., “robust,” “intelligent,” “novel”) without evidence.

**Our Response:**

We thank the reviewer for pointing out these important issues. In the revised manuscript, we carefully addressed all writing, grammar, and consistency concerns as follows:

- The manuscript was re-edited in full, with broken or ungrammatical sentences revised for clarity and readability. For example, the earlier phrase “this situation significant security risks” has been corrected to “this situation poses significant security risks.”
- We have resolved the inconsistencies between ResNet-18 and ResNet-50. The final version consistently states that ResNet-50 is the backbone model of the proposed framework, while ResNet-18 appears only in baseline comparisons.
- The abstract, methodology, and contribution list have been revised for coherence, and all now consistently reference ResNet-50 as the backbone of the proposed model.
- We carefully reviewed the manuscript to reduce excessive use of terms such as “robust,” “intelligent,” and “novel.” These descriptors are now used only when directly supported by experimental results.

Response to Reviewer 2 Comment (Misplaced in R1 Version):

We appreciate the reviewer’s comprehensive feedback highlighting ten critical aspects requiring clarification and technical strengthening. Although these comments were mistakenly placed under Reviewer 2.

*The core claim is a “hybrid CLD-Net” combining YOLOv5 with Faster R-CNN. Similar ensembles and late-fusion detectors are already well explored. You must (i) articulate what is technically new (e.g., a specific fusion rule, calibration scheme, or training curriculum), (ii) explain why this outperforms standard strong baselines (YOLOv8/YOLOv7, EfficientDet, RT-DETR/DETR variants, RTMDet) trained and tuned fairly, and (iii) add a short ablation proving that the proposed fusion (not merely the presence of two models) is responsible for the gains.*

*Method description is incomplete or placeholder-like*

*“Algorithm 1/2” are essentially empty, and “Equations (1)–(20)” are mostly narrative placeholders without definitions. Replace all placeholders with real math (e.g., the exact fusion function \hat{y}=f\left(y_{\mathrm{YOLO}},y_{\mathrm{FRCNN}}\right)y^, confidence reweighting, NMS/Soft-NMS thresholds, IoU thresholds, tie-breaking rules). Provide end-to-end pseudo-code with inputs/outputs, and specify training loss terms, anchors, image sizes, augmentations, and optimizer schedules.*

*Metrics are inappropriate for object detection and not reproducible*

*Reporting “accuracy” for detection is non-standard. Replace or augment with mAP@0.5 and mAP@[.5:.95], per-class AP, AR, precision–recall curves, and F1 at a stated confidence threshold. For speed, report FPS and latency (mean ± std) on specified hardware with batch size and image resolution. Remove the subjective 1–10 “performance/scalability/automation” scores in Tables 2–4; replace with quantitative, reproducible measurements.*

*Dataset provenance, labeling, and splits need rigor*

*You cite a Kaggle “Cotton Leaf Disease” dataset that is primarily classification. If you perform detection, clarify: Did you create bounding boxes? How were they annotated (tool, guidelines)? What was the inter-annotator agreement? Provide the exact train/val/test split (no leakage), stratification rules, augmentation pipeline, and any external data. Without this, the 96.7% figure is not verifiable.*

*Baseline design and fairness are insufficient*

*List all baselines with hyperparameters, training budgets, and model sizes (params/GMACs). Include: single-model YOLOv5 (strongly tuned), Faster R-CNN (tuned), at least one modern one-stage (YOLOv8/RTMDet) and one transformer-based detector (RT-DETR/DETR). Use the same input sizes, epochs, and augmentations where possible. Add ablation studies: YOLO-only, FRCNN-only, naive late fusion vs your proposed fusion; show deltas in mAP and latency.*

*Edge deployment claims lack evidence*

*You claim Raspberry Pi 4 deployability and “95% accuracy,” but there are no concrete measurements. Provide device-level benchmarks: model size, quantization (if any), inference latency per frame, sustained FPS, CPU/GPU/NPU utilization, memory footprint, power draw, and thermal stability over a 10–15 minute run. Include qualitative examples and failure cases from the edge device.*

*Inconsistencies and technical inaccuracies*

*There are contradictions such as using YOLOv5 throughout while the Conclusion says “using the YOLOv8 framework.” Fix all model names consistently. Several definitions around “equations” are incorrect (e.g., describing accuracy as “correct over false positives,” or reusing the same text for different metrics). Audit the entire paper for such errors and correct with standard definitions.*

*Figures, tables, and writing quality need substantial overhaul*

*Many figures are referenced (Figs. 1–11) without visible or technically informative content; captions are generic. Replace with architecture diagrams (with tensor shapes), fusion schematics, PR curves, per-class AP bar charts, latency–accuracy trade-off plots, and qualitative detection visualizations (TP/FP/FN). Table 1 columns like “Power Scheduling/Security/Energy Efficiency” are irrelevant to cotton disease detection—remove or redesign the table to summarize methods, datasets, metrics, and results. Edit the prose for grammar and technical clarity (e.g., “Cotton fleas” → “Cotton is,” avoid phrases like “expatriated object detection,” and remove off-topic mentions such as “drug and vaccine recognition”).*

*Related work and references need curation and credibility*

*Some citations appear peripheral or from questionable venues, while several crucial agricultural-vision and detection papers are missing. Curate to reputable, relevant sources (TPAMI, IJCV, CVPR/ICCV/ECCV, T-ITS, Computers & Electronics in Agriculture, Frontiers in Plant Science, etc.). Discuss recent cotton/plant disease detectors (lightweight YOLO variants, transformer detectors, few-shot domain adaptation, low-light robustness) and clearly position your contribution against them.*

*Reproducibility, statistics, and ethical statements*

*Provide a reproducibility package: code link, configs, random seeds, exact library versions, and trained weights (or a deterministic recipe). Add statistical rigor: report mean ± std over ≥3 runs, confidence intervals, and significance tests where applicable. Include data license compliance, consent (if field images were captured), and a short error analysis and limitations section (e.g., performance under occlusion, early-stage lesions, and domain shifts across fields/cameras).*

*If you address these ten areas with concrete math, proper metrics, fair baselines, real figures, and reproducible evidence the paper will be far stronger and more suitable for a good venue*.
