# Supplementary material for: A distributed framework for zero-day malware detection using federated ensemble models
Source: PLoS One. 2026 Jan 7;21(1):e0339907. doi: 10.1371/journal.pone.0339907 (PMC12779155; doi:10.1371/journal.pone.0339907)
Supplement: Attachment — Submitted filename: Comments to the Author.docx [file pone.0339907.s004.docx]

**Note:** The following comments are based on the revised version of the manuscript submitted in response to the previous reviewers’ feedback.

Comments to the Author

1. Under literature work, the comparison of existing work is not clear, as the example in the first work ref [13] approach is not clear, which deep learning algorithm the authors employed.

2. To make it easier for the readers, it is better if you present a comparison in table form between the proposed approach and existing works based on the limitations of existing work and the improvement of the proposed methodology

3. The dataset collection procedures and tools used to collect the dataset are not clearly explained; they need to be explained.

4. In the article, the authors discussed feature extraction and selection in the proposed method section; however, the paper does not explain the total number of features, which specific features were selected, the criteria used for selection, and the method or algorithm applied for feature selection.

5. The manuscript does not adhere to the required format for PLOS ONE submission guidelines, as example tables and heading styles may need editing.

6. Lastly, recently published studies should be included, and professional language editing is recommended.
