# Supplementary material for: A distributed framework for zero-day malware detection using federated ensemble models
Source: PLoS One. 2026 Jan 7;21(1):e0339907. doi: 10.1371/journal.pone.0339907 (PMC12779155; doi:10.1371/journal.pone.0339907)
Supplement: Attachment — Submitted filename: Responce to Reviewer R3.docx [file pone.0339907.s005.docx]

**A Distributed Framework for Zero-Day Malware Detection Using Federated Ensemble Models**

**Point by Point Response Reviewer 1**

**Comment 1:** The manuscript addresses malware detection using federated learning; however, the authors could clarify how their contributions differ from existing methods. It would be beneficial to articulate the innovative aspects of their approach beyond the standard application of federated learning with ensemble techniques. A more detailed explanation of how their method represents a significant advancement over prior studies would enhance the manuscript.

**Our Response:**

Thank you for highlighting the need to clearly differentiate our contributions from existing techniques. In response, we have revised the end of the Literature Review section to explicitly summarize the limitations of prior studies and to contrast them with the improvements introduced in our proposed method. This new paragraph has been added to provide this clarification:

This hybrid approach is further enhanced by an accuracy-aware weighting technique that prioritizes high-performance nodes to capture evolving malware behavior. A critical comparison of the proposed model against existing techniques highlighting their limitations, drawbacks, and the corresponding improvements of our framework is presented in Table 2.

**Table 2** Comparison of proposed model with existing techniques

| Ref(s) | Existing Limitation | Reason / Drawback | Improvement in Proposed Approach |
| --- | --- | --- | --- |
| [14-19] | Centralized dependency | No privacy, low scalability | Federated, privacy-preserving training |
| [16-17] | Static-only features | Miss dynamic behavior | Multi-modal feature fusion |
| [13-24] | No zero-day evaluation | Seen-only performance | Zero-day adaptive ensemble |
| [30-31] | Non-IID sensitivity | Model drift, instability | Class-specialist local training |
| [32-33] | High communication cost | Large model exchange | Quantized, partial updates |
| [20–25] | Dataset-specific tuning | Poor cross-domain transfer | Cross-dataset generalization |
| [26-28] | Simple aggregation (FedAvg) | Ignores client diversity | Adaptive weighted aggregation |

This **Table** systematically outlines:

- the limitations of each referenced method,
- the underlying reason or drawback, and
- the specific improvement offered by our proposed stacked ensemble federated model.

**Point by Point Response Reviewer 3**

**Comment 1:** Under literature work, the comparison of existing work is not clear, as the example in the first work ref [13] approach is not clear, which deep learning algorithm the authors employed.

**Our Response:** Thank you for your valuable observation regarding the clarity of the literature review, particularly the description of the approach in ref. [13] and the deep learning method used. Your critical analysis helped us identify an important issue: several references were incorrectly linked due to EndNote formatting errors. We sincerely apologies and appreciate your detailed review. We thoroughly re-checked the entire reference list. These have now been corrected, and the updated reference mappings are as follows:

- [8]	→ [9]
- [16] → [17]
- [33] → [25]
- [31] → [26]
- [30] → [27]
- [32] → [28]
- [13] → [14]
- [14] → [15]

In addition, we have revised the sentences associated with the corrected ref. Your valuable observation improves the clarity, accuracy, and consistency of the literature review section, and resolve the concern raised in your comment.

**Comment 2:** To make it easier for the readers, it is better if you present a comparison in table form between the proposed approach and existing works based on the limitations of existing work and the improvement of the proposed methodology

**Our Response:**

Your suggestions are highly valuable, and we truly appreciate them. We have carefully followed your instructions and made the necessary improvements. Thank you for highlighting the need to clearly differentiate our contributions from existing techniques. In response, we have revised the end of the Literature Review section to explicitly summarize the limitations of prior studies and to contrast them with the improvements introduced in our proposed method.

To address this, we added the following explanatory paragraph:

This hybrid approach is further enhanced by an accuracy-aware weighting technique that prioritizes high-performance nodes to capture evolving malware behavior. A critical comparison of the proposed model against existing techniques highlighting their limitations, drawbacks, and the corresponding improvements of our framework is presented in Table 2.

**Table 2** Comparison of proposed model with existing techniques

| Ref(s) | Existing Limitation | Reason / Drawback | Improvement in Proposed Approach |
| --- | --- | --- | --- |
| [14-19] | Centralized dependency | No privacy, low scalability | Federated, privacy-preserving training |
| [16-17] | Static-only features | Miss dynamic behavior | Multi-modal feature fusion |
| [13-24] | No zero-day evaluation | Seen-only performance | Zero-day adaptive ensemble |
| [30-31] | Non-IID sensitivity | Model drift, instability | Class-specialist local training |
| [32-33] | High communication cost | Large model exchange | Quantized, partial updates |
| [20–25] | Dataset-specific tuning | Poor cross-domain transfer | Cross-dataset generalization |
| [26-28] | Simple aggregation (FedAvg) | Ignores client diversity | Adaptive weighted aggregation |

This **Table** systematically outlines:

- the limitations of each referenced method,
- the underlying reason or drawback, and
- the specific improvement offered by our proposed stacked ensemble federated model.

**Comment 3:** The dataset collection procedures and tools used to collect the dataset are not clearly explained; they need to be explained.

**Our Response:**

Thank you for pointing out the need to clearly explain the dataset collection procedures and the tools used. We appreciate this valuable comment. In response, we have added a detailed subsection titled **“4.1 Dataset Creation and Compliance”** that thoroughly describes:

- the data sources,
- the collection tools and environment,
- the filtration and verification process,
- the hashing and metadata extraction tools,
- the labeling procedure,
- and compliance with cybersecurity research standards.

This new section clarifies the entire pipeline from malware acquisition to final dataset construction. The revised text is provided below and has been included in the manuscript.

**4.1. Dataset creation and compliance**

The dataset comprises of 19,600 Win32-type malware samples files accurately collected from various publicly available repositories (e.g GitHub, VirusShare, MalwareBazaar, Malshare, VX-underground). These samples are was collected using standard browser and downloaded as original executable files that are stored in a single repository under snad box environment. This repository is provided as dataset metadata. Additionally, rigorous filtration is applied on this data by computing MD5 and SHA256 hashes using standard python library (hash lib). These files are further verified by VirusTotal that remove files with identical hashes. From this metadata analysis file types (ensure Win32-type executables) and antivirus detection names (signatures) were extracted. To maintain labeling consistency, only files flagged by at least three antiviruses are retained. These files were further verified with classes derived from F-Secure virus detection titles, resulting in 28 different classes for classification. MongoDB is used to store all the malware sample files and metadata that prevent data duplication across dataset. These data creation steps are shown in Figure 2.

**Figure 2:** Dataset Creating Steps

Malware samples were collected and analyzed under the terms and conditions of all approved data source repositories. All the samples analyze in secure, isolated laboratory systems that ensure no malicious files were executed on product systems or shared during outside. The study followed institutional cybersecurity research rules and complied with national malware handling laws.

**Comment 4:** In the article, the authors discussed feature extraction and selection in the proposed method section; however, the paper does not explain the total number of features, which specific features were selected, the criteria used for selection, and the method or algorithm applied for feature selection.

**Our Response:** Thank you for this valuable comment, which helped us improve the clarity of the proposed method. We appreciate your observation regarding the missing details about the total number of extracted features, the specific features used, the selection criteria, and the algorithm applied for feature selection. In response, we have revised the manuscript and added a detailed explanation under the Feature Extraction and Selection subsection.

**4.3. Node-level feature extraction and training**

Feature extraction are important steps for robust classification. To address this, ResNet-18 is employed to extract features at the node level across all nodes. To further compress feature map for optimized computational efficiency Principal Component Analysis (PCA) is employed that eliminate redundant features and reduce 512-dimentional feature vector to 256, while maintaining 90-95% of variance. PCA provide advantage in malware image data due to its unsupervised approach that enable more accurate detection and classification. Additionally, it does not require additional training and label information to preserve variance and to transform correlated features into orthogonal (uncorrelated) components. This enhances the network's ability to learn hierarchical and biased features efficiently.

**Comment 5**: The manuscript does not adhere to the required format for PLOS ONE submission guidelines, as example tables and heading styles may need editing.

**Our Response:**

Thank you for pointing out the formatting issues related to the PLOS ONE submission guidelines. We appreciate this helpful observation. In response, we have carefully reviewed the journal’s formatting requirements and corrected all relevant components in the manuscript.

**Comment 6**: Lastly, recently published studies should be included, and professional language editing is recommended.

**Our Response:**

Thank you for this valuable recommendation. In response, we have thoroughly revised the entire manuscript to ensure professional academic language and clarity. Additionally, we incorporated recently published studies (2024–2025) into the Literature Review ensure that the manuscript reflects the latest advancements in malware detection, federated learning, and lightweight deep learning models.
